# Study of the Efficacy of Probiotic Bacteria to Reduce Acrylamide in Food and In Vitro Digestion

**DOI:** 10.3390/foods11091263

**Published:** 2022-04-27

**Authors:** Siu Mei Choi, Ling Yang, Yuxuan Chang, Ivan K. Chu, Naiping Dong

**Affiliations:** 1 Faculty of Science and Technology, Technological and Higher Education Institute of Hong Kong (THEi), Hong Kong 999077, China; 2 School of Biological Sciences, The University of Hong Kong, Hong Kong 999077, China; u3558930@hku.hk (L.Y.); yxchang@connect.hku.hk (Y.C.); 3 Department of Chemistry, The University of Hong Kong, Hong Kong 999077, China; ivankchu@hku.hk (I.K.C.); naiping.dong@hotmail.com (N.D.)

**Keywords:** probiotic bacteria, acrylamide reduction, biscuits, potato chips, in vitro digestion

## Abstract

In this study, probiotic bacteria as a new post-processing approach to reduce acrylamide (AA) was investigated. The AA reduction ability of selected *Lactobacillus* strains and *Bifidobacterium* strains was demonstrated in (a) AA chemical solutions; (b) food matrices (biscuits and chips) and (c) in vitro digestion. The findings showed tested bacteria exhibited AA reduction ability which was probiotic strain-, AA concentration-, probiotic concentration-, incubation time- and pH-dependent. *L. acidophilus* LA 45 and *B. longum* ATCC 15707 (10^9^ CFU/mL) showed the highest AA reduction (86.85 and 88.85%, respectively) when exposed to 350 ng/mL AA solution for 8 h. The findings also demonstrated that AA reduction ability of selected probiotic strains was pH- and food matrix-dependent in both food matrices (9.45–22.15%) and in vitro digestion model (10.91–21.29%). This study showed probiotic bacteria can lower AA bioaccessibility under simulated digestion.

## 1. Introduction

Dietary exposure to acrylamide (AA) arose as a public health concern. AA is one of the most common process-induced toxicants that is formed when foods, especially those with high carbohydrates and rich in asparagine, are processed at a high temperature of 120 °C or above in the presence of reducing sugar in Maillard browning reaction [1,2,3]. According to the International Agency for Research on Cancer (IARC), AA has been classified as a group 2A, probably carcinogenic to humans.

Based on the Hong Kong First Total Diet Study [4], AA was found in most food groups and makes it impossible to eliminate AA consumption completely. The report showed that potato chips were found to contain the highest level (mean: 680 µg/kg) and cereal-based products such as biscuits contain a mean AA level of 150 µg/kg. They are the major food groups containing AA. For example, the AA content in potato chips is ranging from 430–1100 µg/kg. The dietary exposure to AA was estimated using the local food consumption data and concentrations of AA in food from the local market. The margin of exposure (MOE) is defined as the ratio of BMDL_10_ from animal study to the estimated dietary exposure to AA of the local population, and indicates the health concern level without actually quantifying the risk. The higher the MOE, the lower the health concern. For genotoxic carcinogens, MOE exceeding 10,000 based on BMDL10 from animal study would be of low concern. According to the First Hong Kong Total Diet Study Report in 2013 [4], dietary exposures of the Hong Kong population to AA in adults on average were 0.21 µg/kg bw/day and for high consumer (P95) was 0.54 µg/kg bw/day. MOE was calculated as 857 using the BMDL_10_ for Harderian gland tumors in male mice (0.18 mg/kg bw/day). The current MOE suggested that there is health concern among the local population because of the relatively low MOE values. This may indicate human health concern because of the relatively low figures for a genotoxic carcinogen, while this estimated exposure extent was at the lower end compared to other countries [4]. Hence, it is necessary to explore different possible ways for reducing the AA level in food.

Different approaches have been investigated to reduce AA content in foods, including pre- or post-processing strategies. Most of the methods used for the reduction in AA content in food products mainly focus on steps before and/or during processing. The mitigation steps include changing raw materials with reduced asparagine and/or glucose content and formulations or changing in process conditions and/or technologies such as changing of time and/or temperature of heating or adding appropriate food additives such as antioxidants. These methods only reduced AA formed during food processing. However, these methods did not reduce AA that was produced and some of them may have a negative impact on both taste and appearance of the products.

Probiotic bacteria approach is one of the possible methods due to their ability to decrease the contents of toxic and harmful substances and reduce their bioavailabilities in vitro. The use of probiotic bacteria can be an alternative post-processing approach to reduce concentrations of AA that have already been formed in food products. Lactic acid bacteria (LAB) are of special interest as many of them are “Generally Recognized as Safe” (GRAS) and have been observed to remove toxins and heavy metals [5]. Lactic acid bacteria have been used in various food applications and the probiotic strains of interest can be found in common food products. A study adopting comprehensive strains of LAB demonstrated their capability in reducing AA in vitro [6].

The aim of this study is to investigate the use of five selected probiotic bacteria on reducing AA in some specific food matrices and in vitro digestion models. The findings will provide additional information about AA reduction capacity of probiotic bacteria under various factors including bacterial strains, bacterial and AA concentration, incubation time and pH. It would be of great value in the help of consumers and the food industry to make efforts to reduce AA levels in food and in diet.

## 2. Materials and Methods

### 2.1. Probiotic Strains and Culture Preparation

*Bifidobacterium longum subsp. longum* (ATCC 15707) (*B. longum*), *Bifidobacterium animalis subsp. lactis* (ATCC 700541) (*B. animalis*), *Lactobacillus acidophilus LA45* (PTA-6749) (*L. acidophilus*), *Lactobacillus rhamnosus* (ATCC 53103) (*L. rhamnosus*) and *Lactobacillus casei Shirota* (*L. casei*) isolated from a commercial probiotic beverage (Yakult) were used. All cultures were activated in MRS broth (De Man, Rogosa and Sharpe; Sigma-Aldrich, St. Louis, MO, USA). Subsequently, three subcultures were performed prior to the experiment. For each subculture, an aliquot from the last subculture (4 mL, in MRS broth) was added to 100 mL of sterile MRS broth and incubated for 36 h at 37 °C to achieve maximum growth. The growth curves and bacterial counts by plate count method at the maximum growth of each strain were determined (Figure S1—Supplementary Material).

The bacteria were collected by centrifugation at 2100× *g* for 10 min and washed twice with sterile phosphate buffer saline (PBS) (Sigma-Aldrich, St. Louis, MO, USA). The pellets were re-suspended in sterile PBS to obtain the primary working cultures (10^9^ CFU/mL). To suit the purpose of each experiment, the primary working cultures were diluted to 10^7^ or 10^8^ CFU/mL in sterile PBS. All prepared working cultures were stored at 4 °C.

### 2.2. Reagents

Methanol, HCl (1M), NaOH (1M), NaCl, KCl, NaHCO_3_, NaH_2_PO_4_, Na_2_SO_4_, KSCN, CaCl_2_ * H_2_O, NH_4_Cl, %), KH_2_PO_4_, MgCl_2_, urea, uric acid, mucin, BSA, pepsin, pancreatin, lipase, α-amylase, bile. All reagents were of analytical grades and all organic solvents were of LC/MS HPLC-grade, unless otherwise stated. Acrylamide standard (>99.5%) and ^13^C_3_-acrylamide as internal standard (500 mg/L in acetonitrile) were purchased from Chem Service Inc. (West Chester, PA, USA), and Sigma-Aldrich (St. Louis, MO, USA), respectively. Oasis HLB cartridge (200 mg, 6 cc) was purchased from Waters Corporation (Milford, MA, USA) and Bond Elut Accuat cartridge (200 mg, 3 cc) was purchased from Agilent Technologies, Inc. (Santa Clara, CA, USA)

### 2.3. Preparation of Acrylamide Working Solutions

The AA standard stock solution (1000 μg/mL) and ^13^C_3_-AA internal standard stock solution (40 μg/mL) were prepared. A five-point calibration curve was constructed using the AA working solutions (100 to 500 ng/mL). Each AA working solution was prepared by diluting the stock solutions with Milli-Q water and spiked with ^13^C_3_-AA (200 ng/mL). Various concentrations of AA chemical solutions (350, 750, 1250, 7500 ng/mL) were prepared for the experiments in AA reduction ability. All standard solutions were prepared and stored at 4–8 °C.

### 2.4. Preparation of Digestive Fluids

Digestive fluids used in the in vitro digestion were prepared following the method of Versantvoort et al. [7]. The compositions are shown in Table S1—Supplementary Material. The pH of each digestive fluid was adjusted by HCl (1 M) or NaOH (1 M), when necessary. All digestive fluids were kept at 4–8 °C.

### 2.5. Acrylamide Reduction Ability in Chemical Solutions

In the first stage of the experiment, probiotic strains were incubated with AA chemical solutions under different parameters. The effects of probiotic strain (*B. longum, B. animalis*, *L. acidophilus*, *L. rhamnosus* and *L. casei*), AA concentration (350, 750, 1250 ng/mL), probiotic concentration (10^7^, 10^8^, 10^9^ CFU/mL), incubation time (2, 4, 6, 8 h) and pH (2.5–3.0, 6.5–7.0, and 10.5–11.0) on AA reduction abilities were studied.

#### 2.5.1. Effects of Probiotic Strain, Acrylamide Concentration and Probiotic Concentration

Five probiotic strains at three concentrations (10^7^, 10^8^, or 10^9^ CFU/mL) were studied. The concentration of 10^9^ CFU/mL was close to that of commercial probiotic beverages and yoghurts. Three concentrations of AA (350, 750, and 1250 ng/mL) were also studied. The concentrations were in the range of the AA contents of biscuits and potato chips products in Hong Kong, which are 32–2100 and 160–3000 ng/g, respectively [8].

In this experiment, working cultures of *B. longum*, *B. animalis*, *L. acidophilus*, *L. rhamnosus* and *L. casei* (10^7^, 10^8^, or 10^9^ CFU/mL) or PBS solution (control; pH 6.5–7.0) were added to AA chemical solutions (350, 750, or 1250 ng/mL). The mixtures were briefly vortexed and then incubated at 37 °C for 4 h (close to the total incubation time of the in vitro digestion model) with gentle rotation (55 rpm). After incubation, the mixtures were centrifuged at 20,000× *g* for 10 min at 25 °C. The content of AA in supernatant was determined by LC-MS analysis.

#### 2.5.2. Effects of Incubation Time

The experimental procedures were the same as those described in Section 2.5.1, except that the mixtures of probiotic and AA chemical solutions were incubated for 2, 4, 6 and 8 h at 37 °C.

#### 2.5.3. Effects of pH

The experimental procedures were the same as those described in Section 2.5.1, except that pH of the mixtures of probiotic were adjusted to three pH conditions (2.5–3.0, 6.5–7.0, and 10.5–11.0). The resultant probiotic concentration remained at 10^9^ CFU/mL, and the AA chemical solution of 750 ng/mL was added to the mixture and incubated at 37 °C for 4 h.

### 2.6. Acrylamide Reduction Ability in Food Matrices

To study the effects of food matrices and the effects of pH in different food matrices on AA reduction, the probiotic bacteria (*L. acidophilus* or *B. longum*) were incubated with two selected food samples: Sample 1 (biscuits) and Sample 2 (chips) at three different pH conditions. These two selected food samples (biscuits and potato chips) were found to have relatively high AA content as AA is the common processing-induced contaminants formed during the baking and frying production process of biscuits and chips. Both food samples were spiked with AA (750 ng/g food) chemical solution and internal standard to ensure the final levels of AA were detectable by LC-MS. The mean recoveries of two food matrices were determined (Table S2—Supplementary Material). The relative change of AA content before and after incubation with probiotic bacteria was compared.

Spiked homogeneous food sample (1.0 g) and 0.5 mL of working cultures of *B. longum* and *L. acidophilus* (10^10^ CFU/mL) or PBS solution (control) (all at pH 6.5–7.0), were added to 4.5 mL of sterile PBS solution which was adjusted to different pH with HCl (1 M) or NaOH (1 M) prior to the experiment. The resultant probiotic concentration remained at 10^9^ CFU/mL. The mixtures were then briefly vortexed and incubated at 37 °C for 4 h with gentle rotation at 55 rpm. After incubation, the mixtures were centrifuged at 20,000× *g* for 10 min at 25 °C. The resulting supernatants were cleaned-up by solid phase extraction and subjected to LC-MS analysis to determine AA content.

### 2.7. Acrylamide Reduction Ability under In-Vitro Digestion Model

The efficacy of probiotic bacteria to reduce the AA content in food matrices under a simulated digestion condition using in vitro digestion model described by Versantvoort et al. [7] was determined. The AA content with or without probiotics at the end of the digestion process was compared. The digestion model consisted of three compartments, namely the mouth compartment, stomach compartment and small intestine compartment (Figure S2—Supplementary Material). The samples for in vitro digestion were prepared in two independent groups. The reaction of one group stopped after the stomach compartment whereas it continued for the other group to the small intestine compartment. The boluses (chyme) obtained from the two groups were used to study the efficacy of probiotics to reduce the AA content after digestion in the stomach and small intestine, respectively. The food samples were spiked with AA (7500 ng/g food) to ensure that the final levels of AA were detectable even after the dilutions caused by the in vitro digestion process and compared the relative change of AA content with or without probiotic bacteria. The resultant probiotic concentration remained at 10^9^ CFU/mL. The digestion began with adding 1 g spiked food sample to 5 mL of PBS (control) or cultures of *L. acidophilus* or *B. longum*.

Digestive fluids were added according to the volume ratio of 1 chemical solution/food: 1.3 saliva: 2.6 gastric juice: 2.6 duodenum juice: 1.3 bile: 0.44 NaHCO_3_. All digestive fluids were adjusted to 37 °C before use. Specifically, 1.3 mL of saliva was added to the mixture and the mixtures were incubated at 37 °C and 55 rpm for 5 min in a shaking incubator. This was followed by the addition of 2.6 mL of gastric juice and adjusting the pH of the mixtures to 2.5–3.0 with 1M HCl or NaOH. The mixtures were then incubated at 37 °C and 55 rpm for 2 h. Finally, 2.6 mL duodenum juice, 1.3 mL bile and 0.44 mL NaHCO_3_ (1M) were added simultaneously, and the pH was adjusted to 6.5–7.0 with 1M HCl or NaOH. Again, the mixtures were incubated at 37 °C and 55 rpm for 2 h. At the end of the digestion process (stomach or small intestine compartment), two aliquots from the upper part of the chyme were centrifuged at 20,000× *g* for 10 min at 25 °C. The resulted supernatants were cleaned-up by solid phase extraction and subjected to LC-MS analysis to determine AA content.

### 2.8. Solid-Phase Extraction

To extract AA from the food matrix, two different cartridges were used for solid phase extraction. Oasis HLB cartridge was pre-conditioned with 3.5 mL of methanol followed by 3.5 mL of Milli-Q water, while Bond Elut Accuat cartridge was pre-conditioned with 2.5 mL of methanol and 2.5 mL of Milli-Q water, sequentially. After sample treatment, supernatants were collected and 1.5 mL of clear supernatant were loaded onto the HLB cartridge, followed by washing with 0.8 mL Milli-Q water (all discarded). Then the AA was eluted with 3 mL 50% methanol from HLB cartridge to the Bond Elut Accuat cartridge. Finally, 25 μL of ^13^C_3_-AA (40 ng/mL, internal standard) was added to the 3 mL of effluents and then filtered through a 0.45 μm filter into a glass LC-MS vial for the subsequent LC-MS analysis.

### 2.9. LC-MS Quantification of Acrylamide

The AA analysis was performed with an Agilent 1260 Infinity II HPLC system coupled to an Agilent 6120 Single Quad MS system (Agilent Technologies Inc., Santa Clara, CA, USA) with an electrospray type ionization source. The column of the HPLC system used was a Restek Ultra AQ C18 column (3 μm, 100 × 2.1 mm) (Bellefonte, PA, USA). The sample was separated by the mobile phase (aqueous 0.2% acetic acid and 1% methanol) for 7 min at 0.200 mL/min with 1 min post-time to equilibrate the column [9]. The sample injection volume was 10 μL. The column oven temperature was set at 35 °C. The electrospray was operated in positive ion mode. The conditions used in the ionization source were: 250 °C at 12.0 L/min for the drying gas (N_2_), a nebulizer pressure of 35 psig and a capillary voltage of 3000 V. AA was determined using the Selective Ion Monitoring mode (SIM), monitoring the ions *m/z* 72.0 for AA and 75.0 for ^13^C_3_-AA (internal standard).

By using a five-point calibration curve, the concentration of AA in control and samples were determined and then the percentage of AA reduction was obtained using the following equation.

(1) $$AA\;reduction\;\left(\%\right)=\frac{Conc.\;of\;AA\;in\;control\;\left(PBS\right)-Conc.\;of\;AA\;in\;sample}{Conc.\;of\;AA\;in\;control\;\left(PBS\right)}\%$$

### 2.10. Statistical Analysis

Experiments were conducted in triplicate. All statistical analysis was performed using Python 3.7 with one-way analysis of variance (ANOVA) and Tukey’s means comparison tests (*p* ≤ 0.05) to determine significant differences in the results.

## 3. Results and Discussion

### 3.1. Acrylamide Reduction by Probiotics in Chemical Solution

The AA reduction ability of probiotic bacteria was first assessed in AA chemical solutions. This would provide an understanding of the effectiveness of each probiotic strain under different parameters (AA concentration, probiotic concentration, incubation time and pH) in a less complicated reaction environment and serve as a screening process for the experimental settings (e.g., probiotic strain and concentration) later used with food samples.

#### 3.1.1. Effects of Probiotic Strains, AA Concentration and Probiotic Concentration

**Figure 1:**
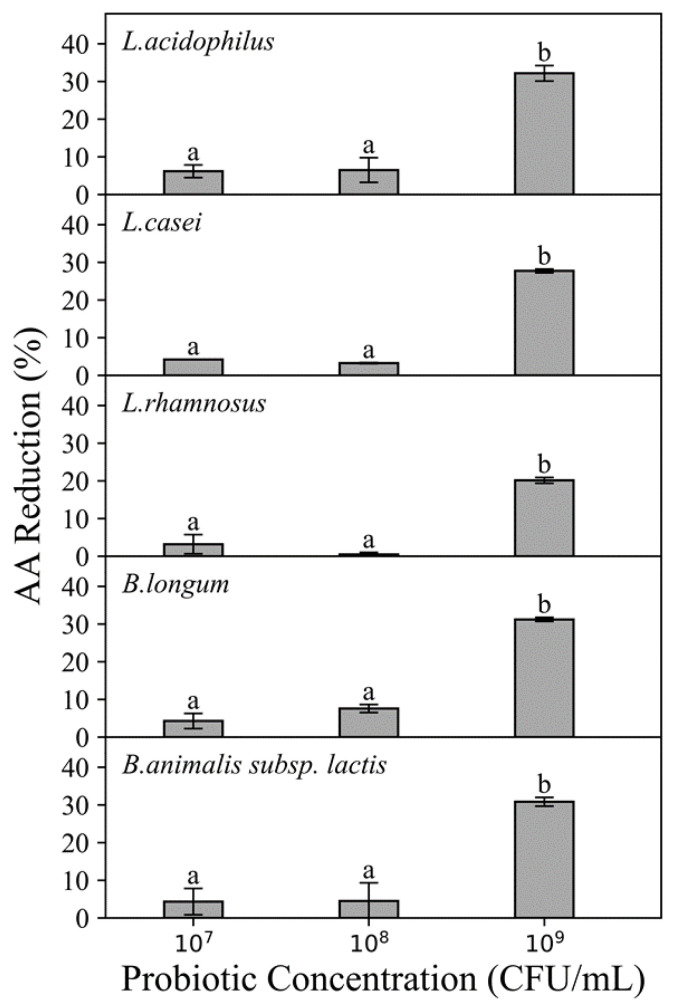
The acrylamide (AA) reduction ability of five strains of probiotic bacteria with concentrations at 10^9^ CFU/mL, 10^8^ CFU/mL and 10^7^ CFU/mL. Exposed to 750 ng/mL AA solution. Incubated at 37 °C for 4 h at pH 6.5–7.0. Expressed as AA reduction percentage. Different characters in same panel indicate significant differences, whereas same character means not.

The percentage of AA reduced by five strains of probiotic bacteria at three different concentrations (10^7^, 10^8^ and 10^9^ CFU/mL) exposed to AA solutions (750 ng/mL) is shown in Figure 1. Effect of probiotic concentration was also observed when compared to the AA reduction percentage (Figure 1). When probiotic concentration increased from 10^7^ to 10^9^ CFU/mL, *L. acidophilus* increased AA reduction percentage from 6.18 to 32.17% whereas *B. longum* increased AA reduction percentage from 4.28 to 31.24% (when exposed to 750 ng/mL AA solution). In general, as the probiotic concentration increased, an increment in AA reduction was observed. For all strains, a significant increase (*p* ≤ 0.05) occurred for strains when the probiotic concentration was raised to 10^9^ CFU/mL. This trend was in accordance with the findings by Rivas-Jimenez et al. [10]. In their study, the AA reduction by *L. casei* Shirota exposed to 50 µg/mL AA solution increased from 12 to 22 µg/mL when probiotic concentration increased from 10^6^ to 10^9^ CFU/mL. In addition, another study concluded that the probiotic concentration was the most important factor influencing aflatoxin removal by *Lactobacillus* strains, among factors including incubation time, temperature and probiotic concentration, and a minimum of 2 × 10^9^ CFU/mL was required to achieve a significant reduction in aflatoxin (5 µg/mL) [11]. This trend supported the binding mechanism to remove toxins, by suggesting that increasing the probiotic concentration increased the number of binding sites, which may eventually lead to a higher reduction.

Based on the above findings, the probiotic concentration of 10^9^ CFU/mL was selected for all following experiments for further investigation as this probiotic concentration is common in commercial probiotic food products and also showed an obvious AA reduction effect.

There are two possible mechanisms to explain the potential toxin-reducing effect. One is the biotransformation of procarcinogens and carcinogens to less toxic metabolites via enzyme and the other is the physical binding by bacterial cell walls. The latter theory is supported, to the best of our knowledge, by major studies devoting to revealing the adsorption mechanisms of AA by LAB, with major binding sites are found at peptidoglycan and teichoic acid structures involving C-O, C=O, and N-H functional groups [6,12,13]. Several factors can influence the binding ability, including LAB species as different species present diverse cell wall composition [6,14], number of bacterial cells and AA concentrations [10,15], and pH of environment [12,15].

**Figure 2:**
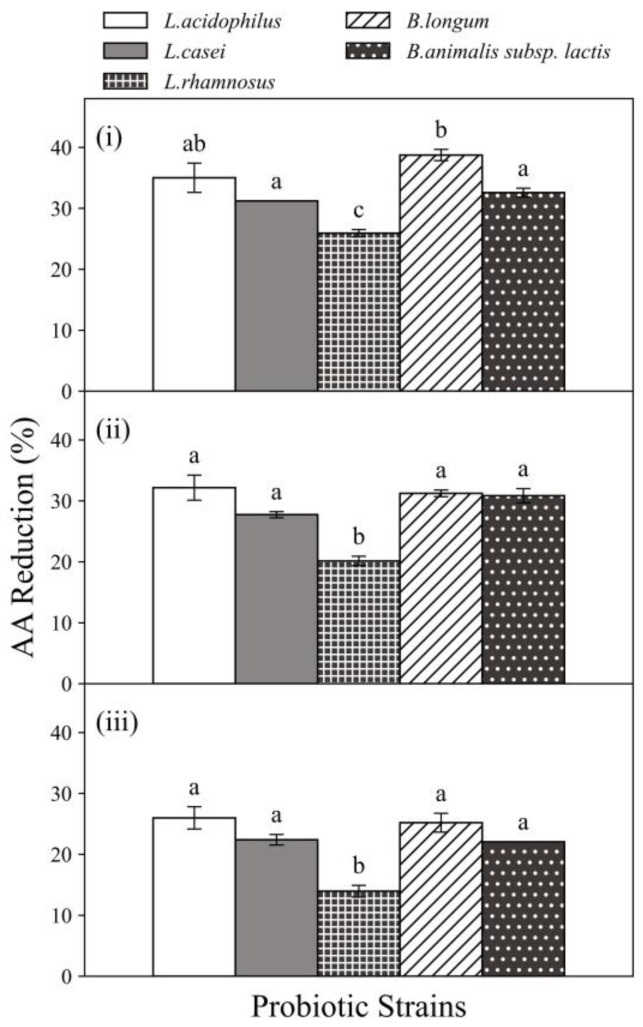
The acrylamide (AA) reduction ability of five strains of probiotic bacteria when exposed to (**i**) 350 ng/mL, (**ii**) 750 ng/mL and (**iii**) 1250 ng/mL AA solutions. Probiotic concentration was 10^9^ CFU/mL. Incubated at 37 °C for 4 h at pH 6.5–7.0. Expressed as AA reduction percentage. Different characters in same panel indicate significant differences, whereas same character indicates not.

As shown in Figure 2, all tested probiotic strains showed ability to reduce AA and the reduction percentage varied when exposed at different AA concentrations. When the AA concentration increased, less percentage of AA was reduced. In particular, at the probiotic concentration of 10^9^ CFU/mL, *L. casei*, *L. rhamnosus* and *B. longum* showed a significant reduction (*p* ≤ 0.05) in their abilities to reduce AA when the AA concentration was increased from 350 to 750 ng/mL and all strains had significantly (*p* ≤ 0.05) declined AA reduction abilities when the AA concentration was further increased to 1250 ng/mL. When exposed to AA concentrations of 350, 750 and 1250 ng/mL, *L. casei* exhibited AA reduction in 31.22%, 27.74% and 22.40%, whereas *L. rhamnosus* and *B. longum* exhibited AA reduction in 25.93%, 20.15% and 13.96% as well as 38.75%, 31.24% and 25.20%, respectively. Similar observations were reported in a previous study of reduction capacities of two *Lactobacillus* strains on AA, that for a defined probiotic concentration (10^9^ CFU/mL), as the solution of AA concentration increased from 10 to 350 µg/mL, the AA reduction percentages decreased from about 80% to 5% [10]. The study has shown that the percentage of AA absorption of *L. plantarum* was 87.05%, which was the highest ability to bind AA. In addition, some strains of lactic acid bacteria have the ability to bind AA and the bind was stable and irreversible [10]. Evidence has shown that toxicant reduction by probiotic bacteria was potentially through a cell wall binding mechanism [12,16,17].

All five probiotic strains exhibited AA reduction abilities at all AA concentrations. Among the five probiotic strains, the highest AA reduction was 38.75 ± 0.92% obtained by *B. longum* (10^9^ CFU/mL) when exposed to 350 ng AA/mL chemical solution incubated for 4 h at 37 °C. Furthermore, all tested strains showed different AA reduction percentages, even at the same AA and probiotic concentration. In general, the current results showed that *L. acidophilus* (35.01 ± 2.40%) and *B. longum* (38.8 ± 0.92%) were more effective at reducing AA among the five selected strains. At probiotic concentration of 10^9^ CFU/mL, significant (*p* ≤ 0.05) differences among the five strains were observed when exposed to AA concentration of 350 ng/mL. This demonstrates the AA reduction ability associated with probiotics are species and strain-specific. These variations may attribute to the differences in the contents of carbohydrates and certain amino acids of the bacterial cell wall, which were reported to be related to the AA binding abilities of probiotic bacteria [12]. Hence, the effect of AA reduction by probiotic bacteria is strain, cell concentration and AA concentration dependent.

#### 3.1.2. Effects of Incubation Time

**Figure 3:**
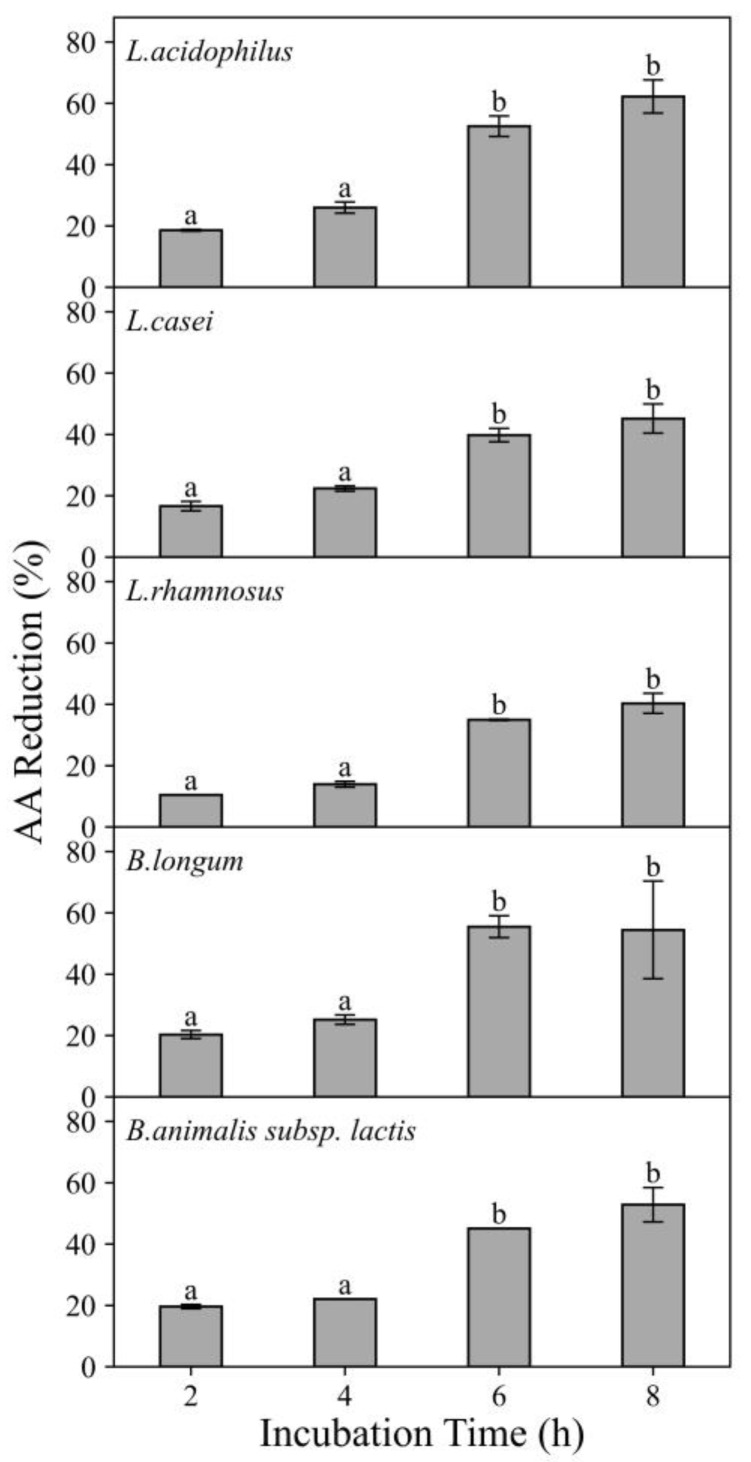
Effects of incubation time on the acrylamide (AA) reduction ability of five strains of probiotic bacteria at 10^9^ CFU/mL when exposed to 750 ng/mL AA solutions for 2–8 h. Incubated at 37 °C at pH 6.5–7.0. Expressed as AA reduction percentage. Different characters in each panel indicate significant differences, whereas same character indicate no significant difference.

The effect of incubation time on AA reduction efficacy of five probiotic strains (10^9^ CFU/mL) exposed to AA chemical solutions (750 ng/mL) is shown in Figure 3. The results revealed that for all strains, from 2 to 6 h, higher AA reduction percentages were achieved with longer incubation time. For example, when exposed to 750 ng/mL AA solution, *L. acidophilus* exhibited AA reduction in 23.01%, 32.17%, 68.27% and 71.99% after 2 h, 4 h, 6 h and 8 h incubation, respectively. When *L. acidophilus* exposed to 350 ng/mL AA solution, it exhibited AA reduction in 29.47%, 35.01%, 79.44% and 86.85% after 2 h, 4 h, 6 h and 8 h incubation, respectively (data not shown). Hence, a significant increase (*p* ≤ 0.05) in AA reduction was found when the incubation time was increased. However, as the incubation time further increased to 8 h, the increment in AA reduction was less apparent. This may indicate that from 2 to 6 h, extending incubation time allowed more binding or enzymatic reactions to occur. However, less binding sites were available or there might be some limiting factors in the activity of acrylamide-degrading enzymes from bacteria after 6 h and therefore the extent of AA reduction increment was lower from 6 to 8 h. The overall increasing trend present in this study was in line with the findings obtained in previous study [15]. The in vitro studies on the AA reduction by *Lactobacillus* strains were conducted and a significant increase occurred for *L. casei* when the incubation time extended from 0 to 4 and 12 h [15].

Among all studied probiotics, *L. acidophilus* and *B.longum* were more effective in AA reduction and therefore they were selected to further study the effects of pH in chemical solution, in the studied food matrices as well as in vitro digestion experiments.

#### 3.1.3. Effects of pH

**Figure 4:**
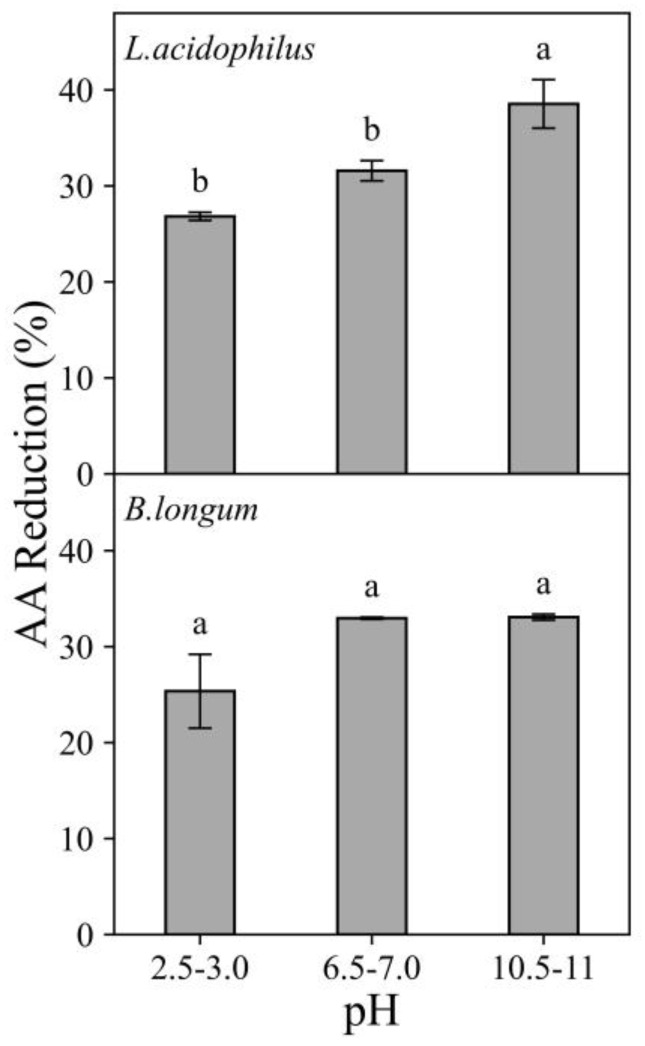
Effects of pH on the acrylamide (AA) reduction ability of *L. acidophilus* and *B. longum* at 10^9^ CFU/mL when exposed to 750 ng/mL AA solutions at three different pH conditions (pH 2.5–3.0, 6.5–7.0 and 10.5–11.0). Incubated at 37 °C for 4 h. Expressed as AA reduction percentage. Different characters in each panel indicate significant differences, whereas same character indicate no significant difference.

The effect of pH over AA reduction ability of *L. acidophilus* and *B.longum* is shown in Figure 4. These two probiotic strains displayed a variable behavior in their AA reduction ability influenced by pH with AA reduction percentage ranging from 25.35 to 38.54% when exposed to 750 ng/mL AA solution (Figure 4). Both probiotic strains showed increasing AA reduction from acidic pH to neutral/ alkaline pH. For *L. acidophilus*, the AA reduction percentage increased significantly from 25.94% to 49.48% with increasing pH from 2.5–3.0 to 10.5–11.0 when exposed to 350 ng AA/mL solution (data not shown). Both *L. acidophilus* and *B. Longum* showed the least effective at acidic pH (2.5–3.0).

Several studies attempted to explain the effects of pH on food toxicant reduction by probiotic bacteria and it appeared the effects were strain-and toxicant-dependent. A study reported that aflatoxin removal by *Lactobacillus* strains was not affected by pH values from 4 to 6 [11]. On the contrary, a study found that increasing in pH from 3 to 8 showed a positive influence on AA reduction by *L. casei* and concluded that the reduction in AA by *Lactobacillus* strains was pH-dependent [15]. In the present study, both strains showed the lowest AA reduction percentage at the acidic pH (pH 2.5–3.0) and this might be explained by the competition for the negatively charged binding sites on bacterial cell wall (e.g., teichoic acid) between AA and protons [6,18].

### 3.2. Acrylamide Reduction by Probiotics in Food Matrices

Two food samples with relatively high AA content were selected from the report of “Acrylamide in Some Popular Foods” by the Hong Kong Centre for Food Safety (CFS) [19]. The selected food products were Sample 1 (biscuits) and Sample 2 (chips), and they were representatives of the major food groups that may contain AA. The differences in AA contents in the two food samples could be explained by the differences in native precursors in food (reducing sugars and asparagine), used additives and/or processing conditions (e.g., cooking time and temperature) [20]. For example, the biscuits had raising agent ammonium carbonates, which may lead to increased AA formation in the product [20].

**Figure 5:**
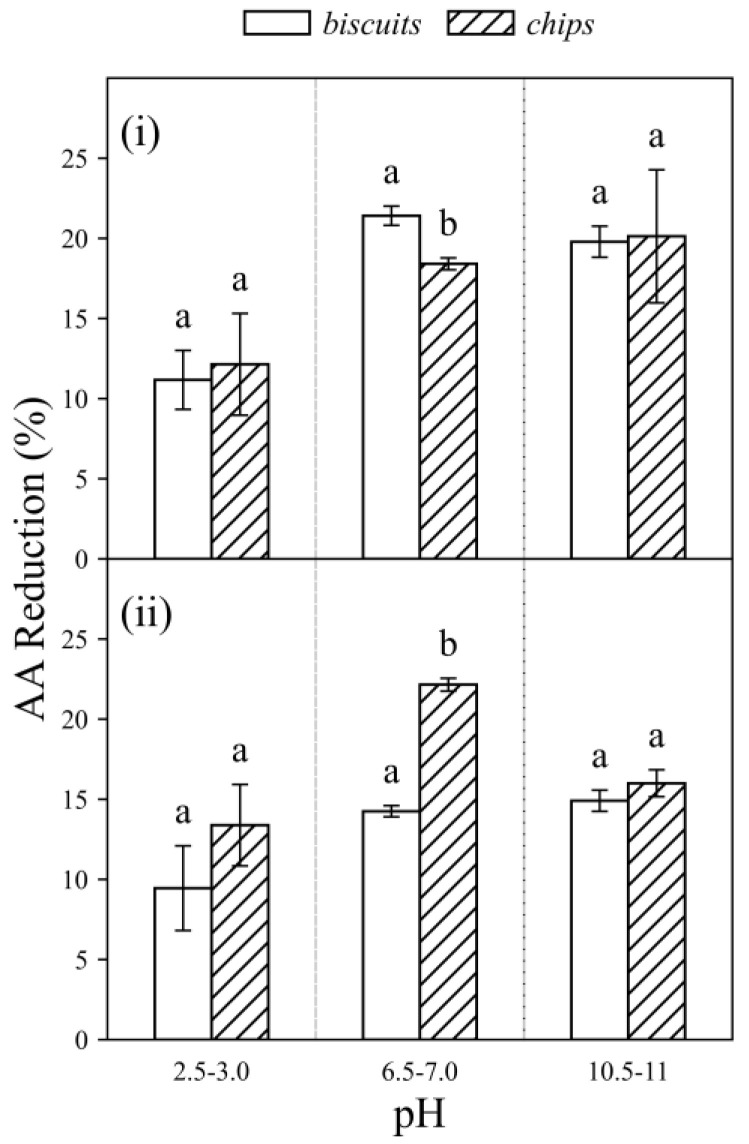
Acrylamide (AA) reduction percentages of (**i**) *L. acidophilus* and (**ii**) *B. longum* (10^9^ CFU/mL) in spiked food samples (750 ng AA/g food): biscuits and chips at three different pH conditions (pH 2.5–3.0, 6.5–7.0 and 10.5–11.0). Incubated at 37 °C for 4 h. Different characters in each panel indicate significant differences, whereas same character indicates no significant difference.

Two probiotic strains, *L. acidophilus* and *B. Longum,* were selected to further examine their ability at reducing AA in food matrices. The strains were incubated with two spiked food samples (biscuits and chips) at concentration of 750 ng AA/g food and three pH conditions. The results are shown in Figure 5.

Regardless of the type of probiotic strains or the pH values, a noticeable reduction in AA was observed (9.45–22.15%) in two different food matrices. In Sample 1 (biscuits) (Figure 5), *L. acidophilus* showed higher AA reduction percentage than *B. longum* especially in pH 6.5–7.0 (21.41 ± 0.60 %) and pH 10.5–11.0 (19.79 ± 0.97 %). However, in Sample 2 (chips) (Figure 5), *B. longum* showed higher AA reduction percentage than *L. acidophilus* at pH 6.5–7.0 (22.15 ± 0.40 %). In terms of the effect of pH, both strains exhibited the lowest reduction percentage at pH 2.5–3.0 which is similar to the findings in AA chemical solutions (Section 3.1.3). When the pH values were raised from acidic pH 2.5–3.0 to 6.5–7.0 and 10.5–11.0, higher AA reduction percentage were found. The results indicated that AA reduction in food matrices could be influenced by probiotic strains, the type of food sample and pH condition. However, further studies on more types of food matrices are required to obtain more in-depth understanding on the effects of food matrices in order to enhance the mitigation strategy to reduce AA in real dietary application.

Previous studies also demonstrated the strain-dependency of AA reduction by probiotic bacteria [10,15] and this may be attributed to variations in bacterial cell wall content [12]. Furthermore, the differences in AA reduction among different food samples by the same strain may be explained by the differences in the ingredients or processing conditions. For example, it was noticed that Sample 1 (biscuits) was added with leavening agent ammonium carbonates which may increase AA formation in the final product [20].

The lowest AA reduction was observed in food samples at pH 2.5–3.0 in all tested conditions. This might result from (i) the reduction in probiotic binding capacity at the acidic pH [21] and (ii) the increase in the amount of AA in food samples at acidic pH. As discussed previously in Section 3.1.3, at pH 2.5–3.0, protons and AA might compete for the negatively charged binding sites on the bacterial cell wall and consequently, the probiotic strain bound to less AA [15]. Besides, a study investigated the kinetics of AA variation along digestion and reported that the gastric pH (2.5–3.0) favored the conversion of AA precursor (e.g., Schiff based formed during processing) in food samples to AA [22]. To confirm the impact of pH on AA content in food products at different pH values, the concentrations of AA in spiked food samples incubated with PBS (control) at different pH were analyzed (Figure S3—Supplementary Material). The result showed that there was an increase in AA content at pH 2.5–3.0 for both food samples when compared with two other pH conditions and this may support the proposed “precursor conversion” phenomenon under binding mechanism.

### 3.3. Acrylamide Reduction by Probiotics in Food Matrices under In Vitro Digestion

In order to investigate the potential application of probiotic bacteria to reduce dietary AA, it is important to understand the impacts of different digestion stages on the reduction mechanism. An in vitro digestion model was used in the current study to simulate the digestive conditions. Two spiked food samples (7500 ng AA/g food) and two probiotic strains (*L. acidophilus* or *B. longum*) were fed into the digestion model and passed three compartments: mouth, stomach and small intestine.

**Figure 6:**
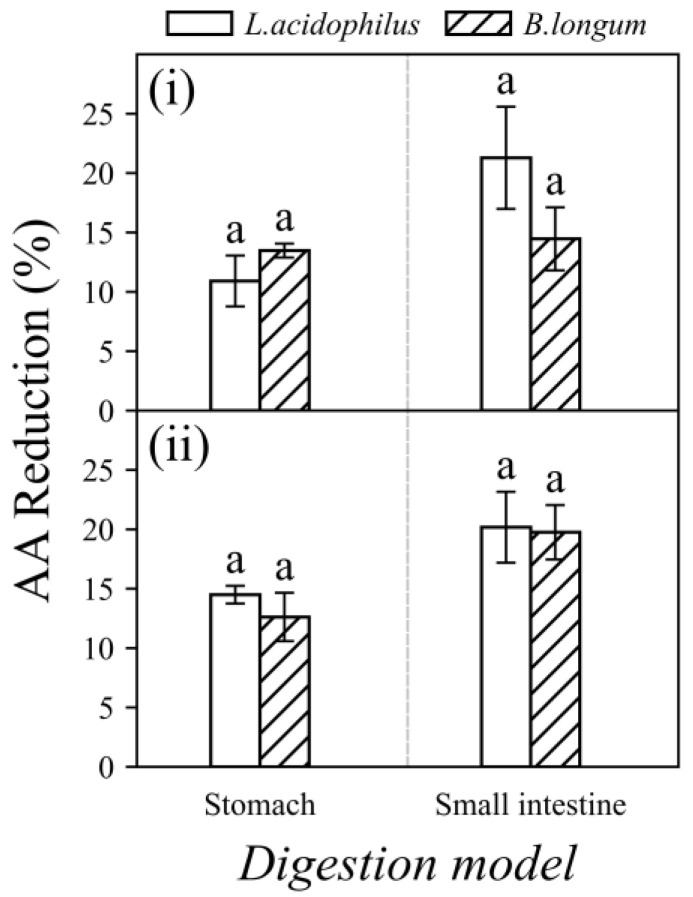
Acrylamide (AA) reduction percentages of *L. acidophilus* and *B. longum* (10^9^ CFU/mL) in spiked food samples (7500 ng AA/g food): (**i**) biscuits and (**ii**) chips after in vitro digestion in stomach and intestinal compartments. Incubated at 37 °C for 4 h. Different characters in each panel indicate significant differences, whereas same character indicates no significant difference.

The percentages of AA reduction by probiotics under the simulated digestive condition are shown in Figure 6. Both *L. acidophilus* and *B. longum* strains displayed AA reduction abilities under the simulated digestive conditions. After gastric digestion, the AA reduction ranged from 10.91 to 14.50% and at the end of intestinal digestion, it was ranging from 14.46 to 21.29%. Both strains resulted in higher AA reduction percentages after intestinal digestion.

The current results showed that the AA reduction in “stomach” (10.91–14.50%) and “small intestine” (14.46–21.29 %) under in vitro digestion is comparable to that in spiked food samples in “pH 2.5–3.0” (9.45–13.38%) and “pH 6.5–7.0” (14.25–22.15%), respectively (as shown in Figure 4 and Figure 6). Hence, it is reasonable to speculate that the increase in AA reduction after intestinal digestion was mainly due to the increase in pH values from the stomach (pH 2.5–3.0) to the small intestine (pH 6.5–7.0) rather than the composition of digestive fluids used in different compartments. This study showed the studied probiotic bacteria can lower AA bioaccessibility under simulated in vitro digestion.

### 3.4. Risk Assessment of Acrylamide after Probiotic Treatment

The current study showed that selected probiotic bacteria can cause AA reduction in food, with percentages ranging from 10–43% depending on the types of probiotic strains and types of food. Margin of exposure (MOE) approach was used for risk assessment. Based on the assumption that the concentration of AA can be reduced by 20–40% on average (with probiotic bacteria), the calculated MOE will be increased to 1071–1428, respectively (using BMDL_10_ of 0.18 mg/kg bw/day). The results also suggest a health concern but at lower level [4]. However, if there was a significant reduction (e.g. 80%) can be achieved by the combination of both pre-processing and post-processing approaches in foods, the MOE would be further increased to exceed 10,000, to further lower the health concern to dietary exposure of AA.

Considering the present findings, this approach opens up a new prospect of reducing the bioaccessibility of AA that can give implication to reduce the risk of AA from our diet. For general consumers, consuming products containing probiotics may serve as a tool to reduce the possible health concerns brought by the dietary AA. For food manufacturers, it is recommended to explore the potential of incorporating probiotics in food products to reduce AA formation during processing and post-processing. Hence, the use of probiotic bacteria for reducing AA level in the studied food matrices (biscuits and potato chips) is a possible option as post-processing approach as demonstrated in this study. Due to the potential synergistic effect, the detoxification effect of multi-strain probiotic formula might provide an alternative approach to reduce toxicants. In our preliminary synergistic study, the combination of *Lactobacillus plantarum* and *Lactobacillus bulgaricus* showed higher AA reduction ability than *Lactobacillus bulgaricus* individually, under three different concentrations of AA chemical standard solution (data not shown). It would be interesting to have further studies following potential synergistic effects of probiotic strains.

## 4. Conclusions

In conclusion, the selected probiotic strains have proved their reduction abilities to reduce AA in chemical solutions, in two selected food matrices (biscuits and potato chips) and in simulated digestive models using these two food matrices. Among the five selected probiotics, *L. acidophilus* and *B. longum* demonstrated the highest reduction ability and they exhibited comparable AA reduction abilities in food matrices (9.45–22.15%) and in food matrices under a simulated digestion condition (10.91–21.29%). AA reduction in food matrices and in vitro digestion might be affected by the type and cell concentration of probiotic strain, food matrix and pH. Hence, probiotic bacteria could be used as an alternative post-processing strategy to reduce AA that was already formed in food products such as biscuits and potato chips.

## Supplementary Materials

The following supporting information can be downloaded at: https://www.mdpi.com/article/10.3390/foods11091263/s1, Figure S1: The growth curves of probiotic strains: *L. acidophilus* (a, ■), *L. casei* (b, ●), *L. rhamnosus* (c, ▲), *B. longum* (d, ▼) and *B.animalis* subsp. *Lactis* (e, ♦). Figure S2: Schematic representation of in vitro digestion model used. Figure S3: Acrylamide (AA) concentration of spiked food sample 1 (biscuits) and food sample 2 (chips) after incubation with PBS solution at three different pH conditions (pH 2.5–3.0, 6.5–7.0 and 10.5–11.0) for 4 h at 37 °C. The food samples were spiked to a concentration of 750 ng AA/g food. Table S1: Constituents of the various synthetic digestion fluids of the in vitro digestion model (per liter). Table S2: The mean recoveries of two food matrices (biscuits and chips).
