# Supplementary material for: Study of the Efficacy of Probiotic Bacteria to Reduce Acrylamide in Food and In Vitro Digestion
Source: Foods. 2022 Apr 27;11(9):1263. doi: 10.3390/foods11091263 (PMC9101460; doi:10.3390/foods11091263)

Supplementary Materials

Figure S1. Supplementary material. The growth curves of probiotic strains: *L. acidophilus* (a, ■), *L. casei* (b, ●), *L. rhamnosus* (c, ▲), *B. longum* (d, ▼) and *B.animalis subsp. Lactis* (e, ♦).

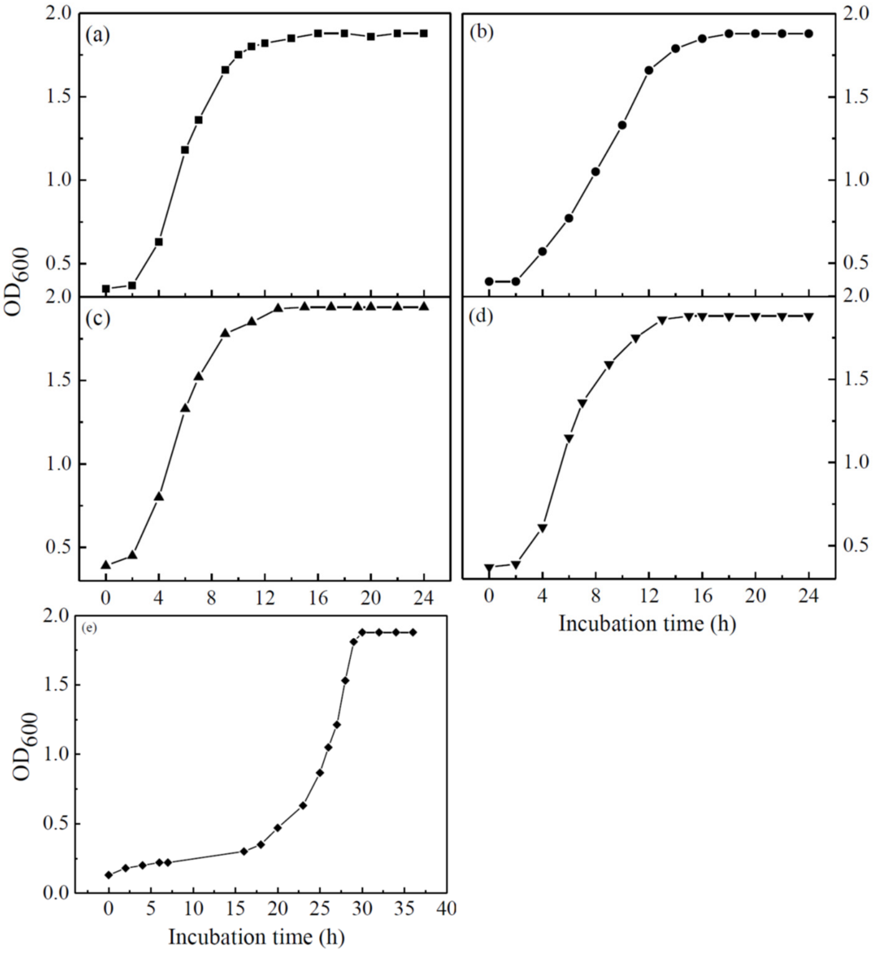

Table S1. Supplementary material. Constituents of the various synthetic digestion fluids of the in vitro digestion model (per liter).

Digestive Juice	Saliva	Gastric Juice	Duodenal Juice	Bile Juice
Inorganic	0.3 g NaCl<br>0.9 g KCl<br>1.7 g $NaHCO_3$<br>0.9 g NaH_2PO_4<br>0.57 g $NaSO_4$<br>0.2 g KSCN	0.82 g KCl<br>0.27 g NaH_2PO_4<br>0.4 g $CaCl_2{\cdot}2H_2O$<br>0.31 g NH_4Cl<br>6.5 mL HCl (37%)	0.56 g KCl<br>3.39 g $NaHCO_3$<br>0.08 g KH_2PO_4<br>0.05 g $MgCl_2$<br>0.18 mL HCl (37%)	0.38 g KCl<br>5.79 g $NaHCO_3$<br>0.15 mL HCl (37%)
Organic	0.2 g urea	0.65 g glucose<br>0.02 g glucuronic acid<br>0.33 g glucosamine hydrochloride<br>0.085 g urea	0.1 g urea	0.25 g urea
Add to the mixture organic + inorganic solution	290 mg α-amylase<br>15 mg uric acid<br>25 mg mucin	1 g BSA<br>2.5 g pepsine<br>3 g mucin	0.2 g $CaCl_2{\cdot}2H_2O$<br>1 g BSA<br>9 g pancreatin<br>1.5 g lipase	0.22 g $CaCl_2{\cdot}2H_2O$<br>1.8 g BSA<br>30 g Bile
pH	7.9 ± 0.2	1.30 ± 0.02	8.8 ± 0.2	8.2 ± 0.2

Table S2. Supplementary material. The mean recoveries of two food matrices (biscuits and chips).

Food matrices	Mean recovery (%)
Sample 1 (biscuits)	97.0 ± 0.1%
Sample 2 (chips)	92.8 ± 0.1%

Figure S2. Supplementary material. Schematic representation of *in vitro* digestion model used.

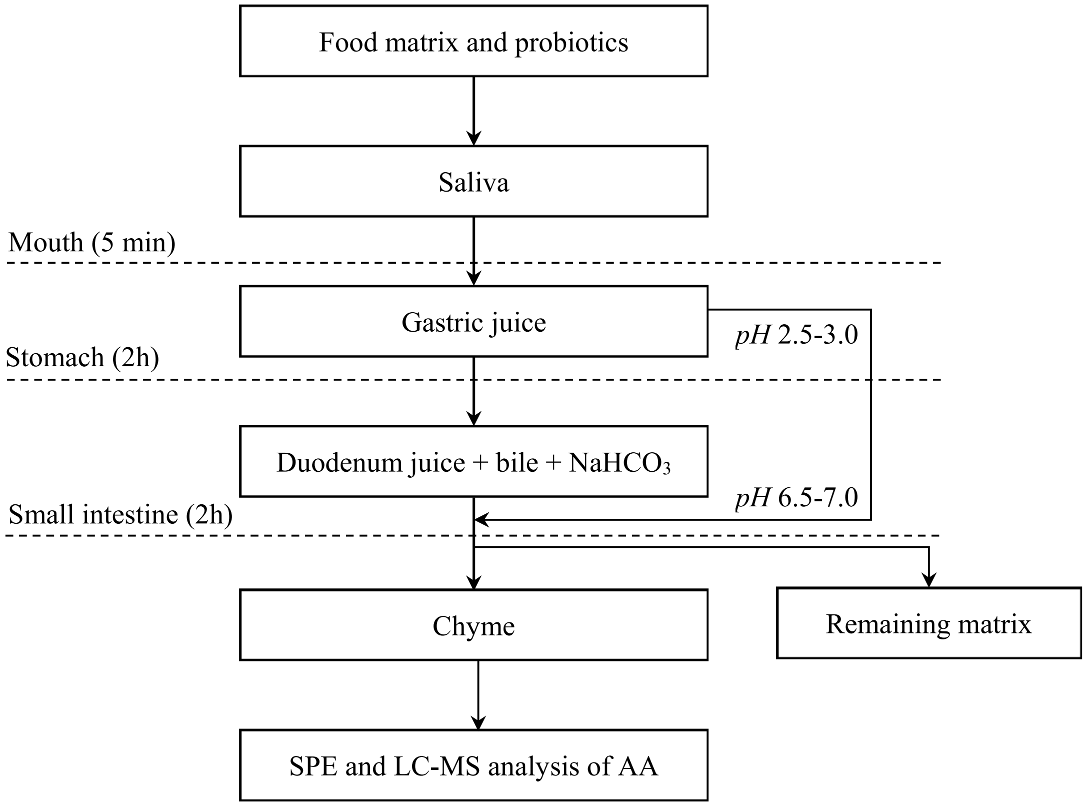

Figure S3. Supplementary material. Acrylamide (AA) concentration of spiked food sample 1 (biscuits) and food sample 2 (chips) after incubation with PBS solution at three different pH conditions (pH 2.5-3.0, 6.5-7.0 and 10.5-11.0) for 4h at 37 ºC. The food samples were spiked to a concentration of 750 ng AA/g food.

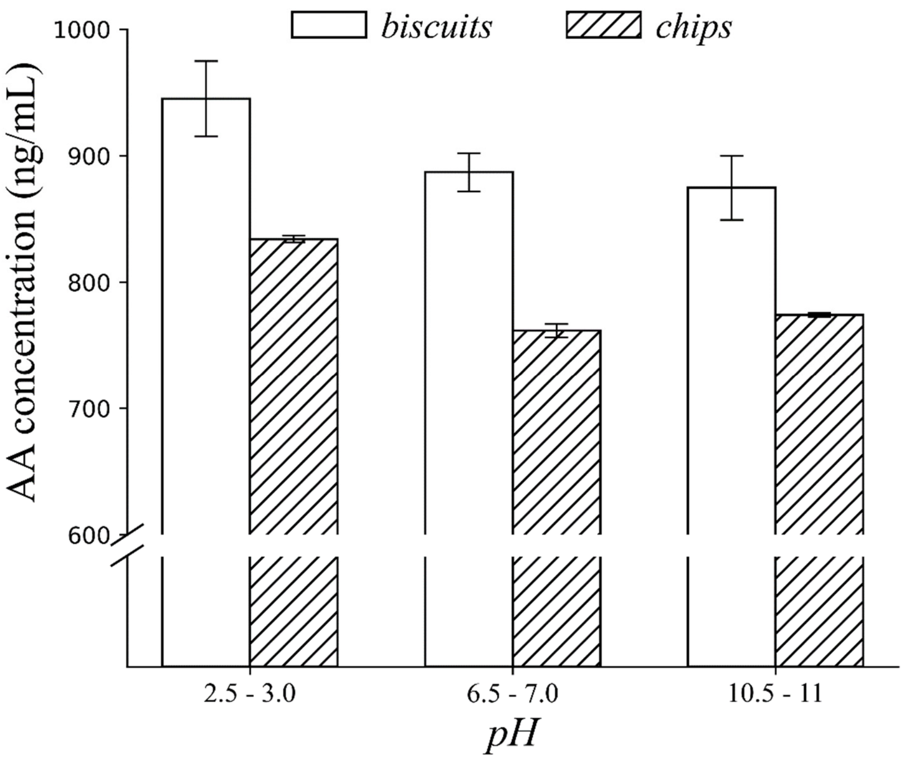

Supplement: Supplementary file 1 [file foods-11-01263-s001.zip › foods-1674218-supplementary.pdf]
